# Bone marrow-derived mesenchymal stem cell-secreted IL-8 promotes the angiogenesis and growth of colorectal cancer

**DOI:** 10.18632/oncotarget.5739

**Published:** 2015-10-23

**Authors:** Jiancheng Wang, Yingnan Wang, Shaochuan Wang, Jianye Cai, Jianqiang Shi, Xin Sui, Yong Cao, Weijun Huang, Xiaoyong Chen, Zijie Cai, Hongyu Li, Adham Sameer A. Bardeesi, Bin Zhang, Muyun Liu, Wu Song, Maosheng Wang, Andy Peng Xiang

**Affiliations:** ^1^ The Biotherapy Center, The Third Affiliated Hospital, Zhongshan School of Medicine, Sun Yat-Sen University, Guangzhou, Guangdong, China; ^2^ Center for Stem Cell Biology and Tissue Engineering, Key Laboratory for Stem Cells and Tissue Engineering, Ministry of Education, Sun Yat-Sen University, Guangzhou, Guangdong, China; ^3^ Department of Gastrointestinal-Pancreatic Surgery, The First Affiliated Hospital of Sun Yat-Sen University, Guangzhou, Guangdong, China; ^4^ Department of Radiology, Third Affiliated Hospital of Sun Yat-Sen University, Guangzhou, Guangdong, China; ^5^ Department of Oncology, The First Affiliated Hospital of Xi'an Jiaotong University Medical College, Xi'an, Shaanxi, China; ^6^ The Cardiovascular Center, Gaozhou People's Hospital, Maoming, Guangdong, China; ^7^ Department of Biochemistry, Zhongshan School of Medicine, Sun Yat-Sen University, Guangzhou, Guangdong, China

**Keywords:** colorectal cancer, mesenchymal stem cells, interleukin-8, angiogenesis

## Abstract

Mesenchymal stem cells (MSCs) have recently been shown to home to tumors and contribute to the formation of the tumor-associated stroma. In addition, MSCs can secrete paracrine factors to facilitate tumor progression. However, the involvement of MSC-derived cytokines in colorectal cancer (CRC) angiogenesis and growth has not been clearly addressed. In this study, we report that interleukin-8 (IL-8) was the most highly upregulated pro-angiogenic factor in MSCs co-cultured with CRC cells and was expressed at substantially higher levels in MSCs than CRC cells. To evaluate the effect of MSC-derived IL-8 on CRC angiogenesis and growth, we used MSCs that expressed small hairpin (interfering) RNAs (shRNA) targeting IL-8 (shIL-8-MSCs). We found that MSC-secreted IL-8 promoted human umbilical vein endothelial cell (HUVEC) proliferation and migration, tube-formation ability and CRC cell proliferation. Additionally, *in vivo* studies showed that MSCs promoted tumor angiogenesis partially through IL-8. Taken together, these findings suggest that IL-8 secreted by MSCs promotes CRC angiogenesis and growth and can therefore serve as a potential novel therapeutic target.

## INTRODUCTION

Colorectal cancer (CRC) is one of the three most common cancers worldwide, with more than 1.2 million new cases, causing approximately 0.6 million deaths per year [1]. The 5-year-survival rate among early-stage CRC patients is approximately 90%, a rate that drops to ~10% in patients with distant metastases [2]. Because angiogenesis is necessary for tumor growth and metastasis, inhibiting tumor angiogenesis is a promising strategy for limiting cancer progression [3].

Angiogenesis, the process of new blood vessel formation from pre-existing vessels, is vital to growth and development [4]. During tumor progression, angiogenesis is an essential pathologic feature of cancer, owing to its important function in ensuring the delivery of oxygen and nutrients to growing tumors [5]. Without an adequate blood supply, a tumor cannot grow beyond 2–3 mm^3^ [6]. Recent research has identified a variety of cytokines that are capable of regulating tumor angiogenesis, including vascular endothelial growth factor (VEGF), fibroblast growth factor (FGF), transforming growth factor beta (TGF-β) and some CXC chemokines [7, 8].

Interleukin-8 (IL-8), also known as CXCL8 or neutrophil-activating protein 1 (NAP-1), is a member of the CXC chemokine family. The basic biological effect of IL-8 is attracting and activating neutrophils [9]. IL-8 in the tumor microenvironment has also recently been implicated in promoting tumor progression. A number of studies have demonstrated that IL-8 participates in cancer cell survival, proliferation and invasion, as well as angiogenesis [9–12]. Endogenously produced IL-8 acts in an autocrine manner to increase the growth of nasopharyngeal tumor spheres and human melanoma cells [10, 11] and also participates in the regulation of patient-derived breast cancer stem cell activity [12]. IL-8 is the first chemokine described to have pro-angiogenic properties, a function that has been confirmed in a variety of tumor types, including ovarian cancer, breast cancer, lung cancer, gastric cancer, and CRC [8, 13–15].

Mesenchymal stem cells (MSCs) are multipotent stromal cells that exhibit self-renewal ability and multipotent differentiation potential [16]. MSCs, which have been the subject of intense investigation, participate in tumor pathogenesis [17]. Accumulating evidence suggests that tumor progression partly depends on interactions between cancer cells and stromal cells [18]. Notably, in this context, bone marrow-derived MSCs can be recruited to tumors, where they become tumor-associated fibroblasts and constitute tumor stroma [19, 20]. It has been established that paracrine signals from cancer-associated stroma have a significant influence on cancer cell behavior [18, 21, 22]. Additionally, MSCs can secrete hundreds of factors, some of which stimulate angiogenesis, such as VEGF, angiopoietin-2, IL-8 and bFGF [23]. However, the mechanism underlying the pro-angiogenic effect of MSCs is still not fully understood.

As noted above, CRC cells can secrete IL-8, but MSCs are also a source of IL-8. Therefore, which source plays a dominant role in tumor progression is an important open question. In this study, we examined the expression of IL-8 in MSCs and CRC cell lines. We found that IL-8 expression in MSCs was substantially higher than in CRC cell lines. Using IL-8-knockdown MSCs to explore the functions of IL-8 secreted by MSCs, we further found that IL-8 secreted by MSCs stimulates CRC angiogenesis and growth *in vitro* and *in vivo*.

## RESULTS

### IL-8 expression is induced in MSCs following interaction with CRC cells

**Figure 1:**
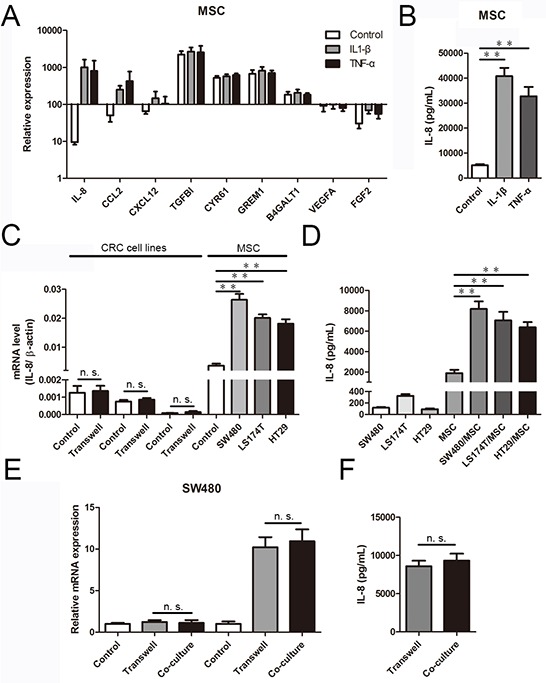
CRC cells induce IL-8 production in MSCs **A.** The expression of pro-angiogenic genes by MSCs treated with recombinant IL-1β (30 ng/mL) and TNF-α (20 ng/mL) were determined using RNA-Seq data. The results are presented as a histogram. **B.** IL-8 protein levels in culture media determined by ELISA in MSCs pretreated with recombinant IL-1β (30 ng/mL) and TNF-α (20 ng/mL) for 36 h. The results are presented as the mean values from three independent experiments (**, *p* < 0.01). **C.** In a CRC cell/MSC transwell system, MSCs were co-cultured with CRC cells (SW480, LS174T and HT29). After 36 h, IL-8 mRNA expression was measured using qRT-PCR and normalized to β-actin mRNA (**, *p* < 0.01). **D.** IL-8 protein levels in culture media determined by ELISA in CRC cells and MSCs before and after co-culture for 36 h. The results are presented as the mean values from three independent experiments (**, *p* < 0.01). **E.** MSCs and SW480 were co-cultured in a transwell system and a direct contact system separately for 36 h, and IL-8 expression in SW480 and MSCs was measured using qRT-PCR. **F.** IL-8 protein levels in culture media determined by ELISA in a transwell system and a direct contact system of MSCs and SW480 for 36 h. The results are presented as the mean values from three independent experiments.

Inflammation is a characteristic feature of the tumor microenvironment [24]; accordingly, we first investigated the influence of inflammatory cytokines on MSCs. To this end, we performed genome-wide RNA-seq to assess the changes in the expression of pro-angiogenic genes related to the angiogenesis of MSCs treated with recombinant IL-1β (30 ng/mL) and TNF-α (20 ng/mL; the global transcriptional profiling data to be published elsewhere). As shown in Figure 1A, MSCs from three different donors expressed a series of angiogenesis-related factors, including CCL2, CXCL12, TGFB1, CYR61, GREM1, B4GALT1, VEGFA and FGF2. Among these genes, the levels of IL-8 expression were increased 106.0-fold and 84.6-fold in MSCs stimulated with recombinant IL-1β and TNF-α, respectively, compared with control cells. Because IL-8 is a chemokine that promotes tumor angiogenesis, we further analyzed the levels of secreted IL-8 protein in MSCs treated with recombinant IL-1β (30 ng/mL) and TNF-α (20 ng/mL) using ELISA. We observed an 8.02-fold and 6.44-fold promotion of IL-8 secretion, respectively (Figure 1B).

Next, we studied the interactions in culture of SW480, LS174T and HT29 human colorectal carcinoma cells with MSCs. As shown in Figure 1C, IL-8 expression was unchanged when CRC cells were co-cultured with MSCs for 36 h. In contrast, IL-8 expression increased in MSCs after co-culture. Notably, IL-8 mRNA expression, normalized to β-actin mRNA, was dramatically different between MSCs and CRC cells, with IL-8 mRNA levels being 21.1–212.2-fold higher in MSCs than in CRC cells. The higher and upregulated IL-8 mRNA levels in MSCs supported the conclusion that MSCs were the main source of IL-8. Furthermore, we measured the secretion of IL-8 in the culture media from CRC cells and MSCs separately. Minimal IL-8 production was observed in the media from pure CRC cells, and markedly higher IL-8 production was observed in the media from pure MSCs. After 36 h of co-culture separated by a transwell membrane, which allows the exchange of soluble factors but prevents direct cell-cell contact, IL-8 levels increased 3.4–4.3-fold compared with untreated MSCs (Figure 1D). Thus, IL-8 was induced in MSCs following interaction with CRC cells, and the secretion of IL-8 in MSCs was substantially higher than in CRC cells. Moreover, to determine whether direct contact had an effect on CRC cell-induced upregulation of IL-8 expression in MSCs, we co-cultured GFP-expressing MSCs with CRC cells in a direct co-culture system or a transwell system. After 36 h of co-culture, GFP-expressing MSCs in the direct contact system were sorted by flow cytometry. Then, the IL-8 expression of each group was determined by quantitative reverse transcription-polymerase chain reaction (qRT-PCR). There was no increase in IL-8 expression in CRC cells after 36 h of co-culture in a direct contact system. In contrast, IL-8 expression in MSCs increased after co-culture in the direct contact system. Notably, compared with the direct contact system, the IL-8 expression levels of CRC cells and MSCs were induced equally in the transwell system (Figure 1E). In addition, ELISAs revealed no marked differences in IL-8 secretion of culture media between the transwell system and the direct contact system (Figure 1F).

### MSC-secreted IL-8 enhances human umbilical vein endothelial cell proliferation

To address the influence of IL-8 on angiogenesis in CRC, we further investigated the effect of IL-8 knockdown on cultured MSCs. Western blotting and qRT-PCR assays indicated that IL-8 protein and mRNA levels were decreased in MSCs transfected with a vector expressing a short hairpin (inhibitory) RNA (shRNA) targeting IL-8 (shIL-8-MSCs), respectively (data not shown).

**Figure 2:**
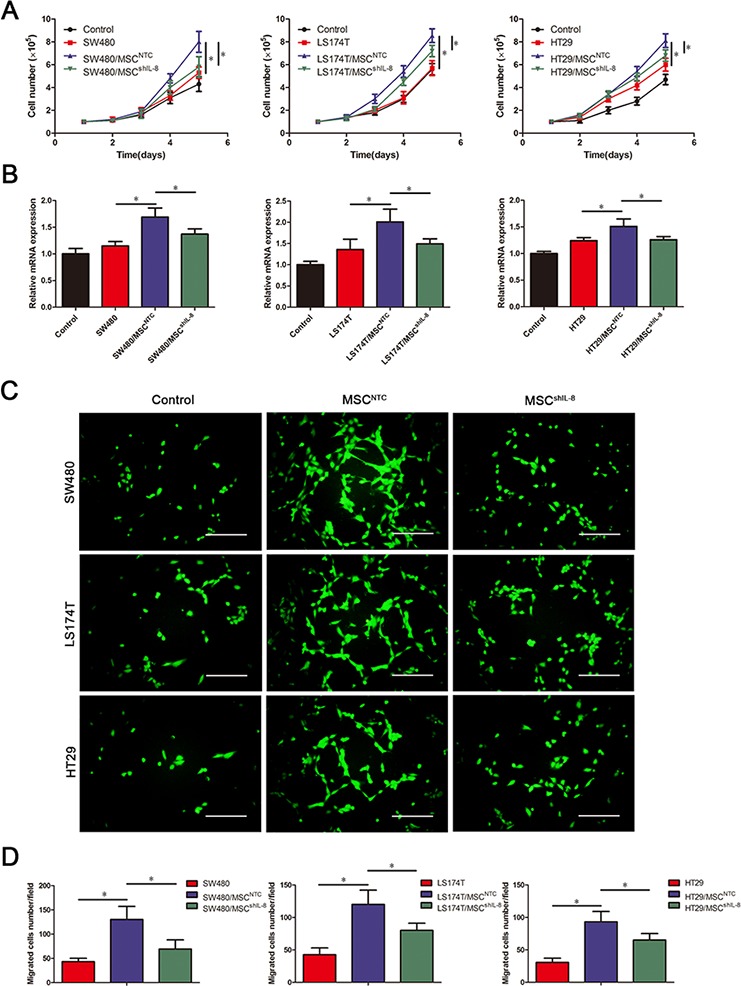
MSCs promote endothelial cell proliferation and migration through IL-8 secretion **A.** The numbers of HUVECs following culture with fresh medium or conditioned medium from CRC cells, CRC cell/MSC co-cultures, or CRC cell/shIL-8-MSC co-cultures were determined every day for 5 days. The cell counts are presented as the mean values from three independent experiments using a hemocytometer (*, *p* < 0.05). **B.** HUVECs were cultured with conditioned medium as indicated in (A), and Ki-67 mRNA expression in HUVECs was measured by qRT-PCR (*, *p* < 0.05). **C.** Assay of HUVEC migration in response to different conditioned media using 8-μm-pore membrane filters. Calcein AM-stained cells indicates HUVECs that migrated through the transwell membrane. Scale bar, 100 μm. **D.** The numbers of HUVECs that passed through the filters was counted. The cell counts are presented as the mean values per field from at least five randomly selected fields from three independent experiments (*, *p* < 0.05).

To ascertain whether IL-8 secreted by MSCs was involved in CRC angiogenesis, we explored the effect of IL-8 knockdown in MSCs on the proliferation, migration, and tube-formation ability of human umbilical vein endothelial cells (HUVECs). To test cell proliferation, we cultured HUVECs in the presence of conditioned medium from CRC cells alone, CRC cell/MSC co-cultures, or CRC cell/shIL-8-MSC co-cultures. Conditioned medium from CRC cells had little stimulatory effect on HUVEC proliferation, whereas conditioned medium from CRC cell/MSC co-cultures effectively increased HUVEC numbers. Notably, conditioned medium from CRC cell/shIL-8-MSC co-cultures promoted a substantially smaller increase in cell numbers compared with the CRC cell/MSC co-cultures (Figure 2A). The mRNA levels of the proliferation marker Ki-67 were consistent with the cell count results (Figure 2B), as were the results obtained using Cell Counting Kit-8 (CCK-8) assays (Supplementary Figure S2A & S2B).

### MSC-secreted IL-8 induces HUVEC migration and tube formation

To determine whether paracrine factors in conditioned medium from CRC cell/MSC co-cultures promoted HUVEC migration, we performed transwell migration assays. These assays revealed that conditioned medium from CRC cells alone promoted the migration of only a small number of HUVECs. Compared with CRC cells alone, conditioned medium from the co-culture groups had a substantially greater effect on HUVEC migration. However, the migration-stimulating effect of conditioned medium from CRC cell/shIL-8-MSC co-cultures was significantly less than that of conditioned medium from CRC cell/MSC co-cultures (Figure 2C & 2D).

To confirm the angiogenesis-promotion function of IL-8, we stimulated HUVECs with recombinant human IL-8 (rhIL-8). As expected, rhIL-8 significantly induced HUVEC proliferation, as assessed by CCK-8 and cell count assays (Supplementary Figure S3A–S3C), and stimulated a remarkable increase in HUVEC migration (Supplementary Figure S3D).

**Figure 3:**
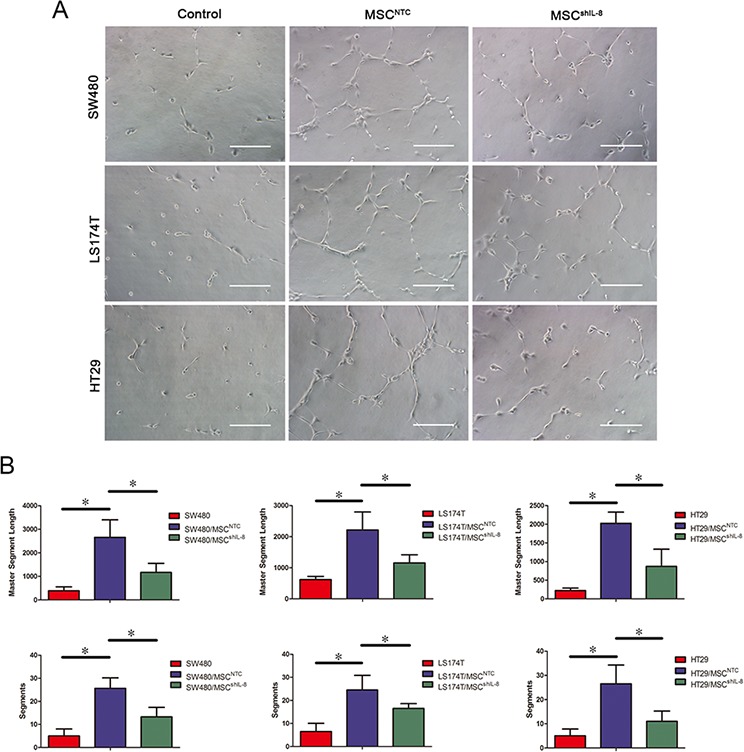
MSCs promote endothelial tube formation through IL-8 secretion **A.** HUVECs were cultured on Matrigel in the presence of conditioned medium from CRC cells, CRC cell/MSC co-cultures or CRC cell/shIL-8-MSC co-cultures for 4 h. The images show the tube-formation ability of HUVECs in different conditioned media. Scale bar, 100 μm. **B.** Capillary-like structures were evaluated by quantifying tube lengths and numbers using the Angiogenesis Analyzer module in the ImageJ toolkit. The results are presented as the mean values per field from at least five randomly selected fields from three independent experiments (*, *p* < 0.05).

Next, we investigated HUVEC tube-formation ability, a functional property of endothelial cells. To this end, we cultured HUVECs on Matrigel in the presence of conditioned medium from CRC cells, CRC cell/MSC co-cultures or CRC cell/shIL-8-MSC co-cultures. Tube formation after a 4 h incubation, imaged by phase-contrast microscopy, is shown in Figure 3A. These assays revealed that conditioned medium of CRC cell/MSC co-cultures enhanced the tube-formation ability of HUVECs, as measured by both the numbers and lengths of tubes formed. Notably, tube-formation ability was decreased using conditioned medium from CRC cell/shIL-8-MSC co-cultures (Figure 3B). In addition, we detected the tube-formation ability of HUVECs after rhIL-8 stimulation. Compared with the control group, the tube-formation ability was increased after rhIL-8 stimulation (Supplementary Figure S3E). Collectively, these results indicate that IL-8 secreted by MSCs stimulates tumor angiogenesis.

### IL-8 secreted by MSCs promotes tumor cell proliferation

**Figure 4:**
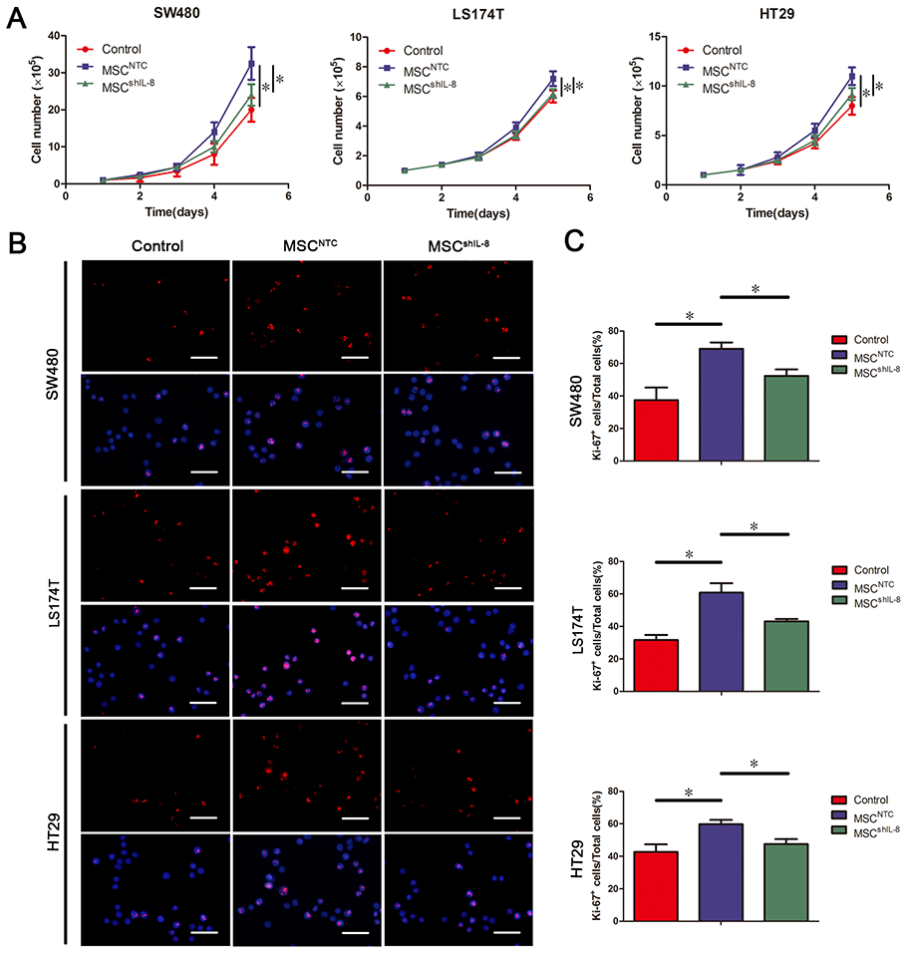
MSC-secreted IL-8 stimulates CRC cell proliferation **A.** The number of CRC cells (SW480, LS174T and HT29) following culture with fresh medium or conditioned medium from CRC cell/MSC co-cultures or CRC cell/shIL-8-MSC co-cultures was determined. The cell counts are presented as the mean values from three independent experiments using a hemocytometer (*, *p* < 0.05). **B.** Cultured CRC cells (SW480, LS174T and HT29) with conditioned medium indicated in (A) were immunostained for the proliferation marker Ki-67 and counterstained with the nuclear dye DAPI. Scale bar, 50 μm. **C.** The number of Ki-67–positive cells was counted, and the percentage of Ki-67–positive cells was calculated. The results are presented as the mean values per field from at least five randomly selected fields from three independent experiments (*, *p* < 0.05).

In addition to assessing tumor angiogenesis, we evaluated the influence of MSCs on CRC cell growth. As shown in Figure 4A, MSCs promoted CRC cell proliferation, as verified by CCK-8 assays (Supplementary Figure S4A & S4B). The expression of proliferating cell nuclear antigen (PCNA) and Ki-67, markers of cell proliferation, was elevated in CRC cells co-cultured with MSCs but was little changed in CRC cells co-cultured with shIL-8-MSCs (Supplementary Figure S4C). Consistent with Ki-67 mRNA expression, immunostaining for Ki-67 yielded similar results (Figure 4B & 4C). In addition, CRC cells grew faster after stimulation with recombinant human IL-8 (Supplementary Figure S5A–S5C). These results indicate that MSC-secreted IL-8 promotes CRC cell proliferation *in vitro*.

### MSCs promote tumor angiogenesis and growth *in vivo*

**Figure 5:**
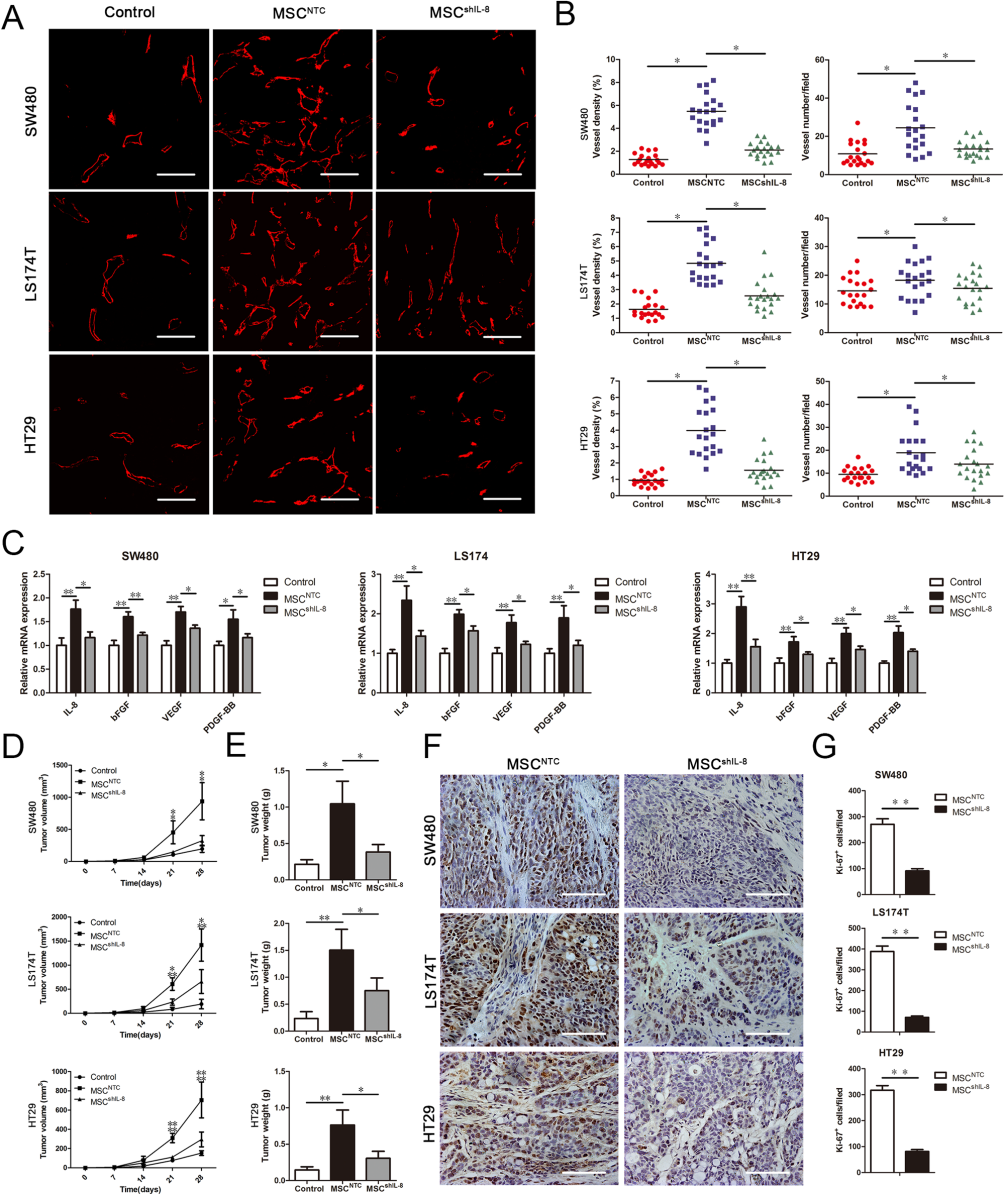
MSCs promote tumor angiogenesis and growth *in vivo* **A.** A total of 2 × 10^6^ CRC cells (SW480, LS174T and HT29) alone or together with 2 × 10^6^ MSCs or shIL-8-MSCs was injected into the flanks of nude athymic mice (*n* = 5). The tumors were harvested after 4 weeks and sectioned. The slides were stained with an anti-CD31 antibody for evaluating vessel morphology. Scale bar, 100 μm. **B.** MVD was determined by measuring the percentage of each field occupied by a CD31-positive signal. The vessel numbers per field were also counted. For each group, four randomly chosen high power fields per tumor (*n* = 5) were analyzed (*, *p* < 0.05). **C.** Total mRNA from tumor tissues was extracted, and the mRNA expression of IL-8, bFGF, VEGF and PDGF-BB in tumor tissues was measured by qRT-PCR (*, *p* < 0.05, **, *p* < 0.01). **D.** Male nude mice (4–6 weeks old) were injected with 2 × 10^6^ CRC cells (SW480, LS174T and HT29) alone or together with 2 × 10^6^ MSCs or shIL-8-MSCs (*n* = 5). The tumors were harvested after 4 weeks. Tumor volume was measured every week and calculated using the formula: tumor volume (mm^3^) = 0.5 × width^2^ × length (*, *p* < 0.05, **, *p* < 0.01). **E.** Tumors were harvested and weighed after sacrifice (*, *p* < 0.05, **, *p* < 0.01). **F.** Paraffin-embedded tumor tissue sections from xenografts were analyzed by immunohistochemistry for the expression of the proliferation marker Ki-67. Scale bar, 100 μm. **G.** Immunohistochemistry staining was analyzed by counts of Ki-67–positive cells in random fields. The cell counts are presented as the mean values per field from at three representative fields at 400x magnification for each tumor (3 tumors per group; **, *p* < 0.01).

To further test whether MSCs display enhanced tumor angiogenic activity *in vivo*, we subcutaneously injected 2 × 10^6^ CRC cells or a mixture of 2 × 10^6^ CRC cells and 2 × 10^6^ MSCs (parental or shIL-8–expressing) into nude mice. After 4 weeks, the tumors obtained from nude mice were further evaluated for vessel numbers and microvessel density (MVD), determined by staining for the endothelial marker CD31 (Figure 5A). As shown in Figure 5B, there was an increase in MVD and vessel numbers in tumors formed from CRC cell/MSC–derived tumors compared with CRC cell–derived tumors. In addition, both MVD and vessel numbers were decreased in CRC cell/shIL-8-MSC–derived tumors compared with CRC cell/MSC–derived tumors. To further investigate the change in IL-8 and other angiogenesis-related factors participating in this process, we detected the mRNA expression of IL-8, bFGF, VEGF and PDGF-BB in tumor slides. We found that the mRNA levels of these angiogenesis-related factors increased in CRC cell/MSC–derived tumors compared with CRC cell–derived tumors. Meanwhile, the mRNA levels of these angiogenesis-related factors were decreased in CRC cell/shIL-8-MSC–derived tumors compared with CRC cell/MSC–derived tumors (Figure 5C). These results demonstrate that IL-8 knockdown results in a decrease in the pro-angiogenic function of MSCs.

To evaluate tumor growth, tumors were measured every week for 4 weeks (Figure 5D), and tumor weights were measured after the mice were euthanized (Figure 5E). Tumors formed from CRC cells co-injected with MSCs were clearly substantially larger than those formed from CRC cells alone, whereas the effect of shIL-8-MSCs on tumor growth was weaker than that of parental MSCs. Furthermore, tumor sections were examined for the expression of Ki-67 by immunohistochemical staining. Representative sections indicated a decrease in the number of Ki-67-positive nuclei (brown stain) in CRC cell/shIL-8-MSC–derived tumors compared with CRC cell/MSC–derived tumors (Figure 5F). Quantification of Ki-67-positive nuclei across all sections revealed that, compared with the CRC cell/shIL-8-MSC–derived tumors, IL-8 knockdown caused a decrease in the number of Ki-67-positive cells in the CRC cell/MSC–derived tumors (Figure 5G). These results suggest that IL-8 secreted by MSCs is an important contributor to tumor growth.

## DISCUSSION

Recent decades have witnessed a growing appreciation of the importance of the contribution of MSCs to the tumor microenvironment [25]. MSCs can modulate immune responses, tumor angiogenesis, metastasis and drug resistance, thereby affecting tumor progression [26]. In CRC, it is well accepted that MSCs can integrate into the tumor architecture, potentially as carcinoma-associated fibroblasts [22]. However, the mechanisms underlying the relationship between CRC cells and MSCs have remained unclear. Our findings provide the first demonstration that MSC-secreted IL-8, rather than that secreted by CRC cells, is mainly involved in stimulating tumor angiogenesis.

Studies have demonstrated that numerous cytokines secreted by MSCs can induce angiogenesis during tumor progression [27–29]. For example, bone marrow-derived MSCs have been shown to migrate towards pancreatic tumor cells and increase the sprouting of HUVECs, a process mainly governed by the pro-angiogenic factor VEGF [28]. In addition, MSC-secreted IL-6 increases cancer cell secretion of endothelin-1 (ET-1), which then stimulates endothelial cells to form new blood vessels [30]. Our *in vivo* data clearly show that MSCs facilitate tumor growth by enhancing angiogenesis via a mechanism that depends on IL-8 secretion. However, MSCs have also been suggested to correlate with the anti-angiogenic process. In a mouse model of human glioma, MSCs have been reported to downregulate the platelet-derived growth factor (PDGF)/PDGF receptor axis in endothelial cells, thereby inhibiting tumor angiogenesis and suppressing tumor growth [31]. It has also been reported that high numbers of MSCs are cytotoxic to endothelial cells, suggesting a context in which MSCs might be an effective anti-angiogenic therapy [32].

It has been well documented that tumors are usually infiltrated by inflammatory cells and inflammatory factors. IL-1β and TNF-α are the most important inflammatory molecules involved in cancer-related inflammation [33]. According to related studies, recombinant IL-1β could induce approximately 100-fold increases of IL-8 expression in MSCs that had been treated for 48 h [34]. In addition, in the conditioned medium of TNF-α-stimulated MSCs, secreted IL-8 was significantly increased approximately 50-fold compared with pure MSCs [35]. In our model of MSC-CRC cell interactions, our focus was on the primary source of IL-8, an issue not specifically addressed in other studies. For this purpose, we evaluated IL-8 mRNA and protein levels in MSCs and CRC cells before and after co-culture. Our data showed that co-culture increased IL-8 mRNA levels in MSCs, but had virtually no effect on IL-8 mRNA levels in CRC cells. In keeping with this, β-actin–normalized IL-8 levels in MSCs were substantially higher than those in CRC cells. ELISAs showed that pure CRC cells secreted minimal IL-8, and pure MSCs secreted substantially more IL-8, indicating that IL-8 secreted by MSCs is dominant in the tumor microenvironment. These findings were further confirmed by knocking down IL-8 in MSCs using a GFP-shRNA construct that interferes with IL-8, which reduced the pro-angiogenic ability of MSCs. Proliferation assays further revealed that conditioned medium from CRC cell/shIL-8-MSC co-cultures was less effective in promoting HUVEC proliferation than conditioned medium from CRC cell/MSC co-cultures. IL-8 knockdown in MSCs similarly reduced the ability of conditioned medium from co-culture to promote the migration and tube-formation ability of HUVECs. We also confirmed that IL-8 is sufficient to produce these effects, showing that stimulation with rhIL-8 induced HUVEC proliferation, migration, and tube formation. To extend these results to an *in vivo* setting, we injected nude mice with CRC cells and MSCs or shIL-8-MSCs and evaluated the angiogenesis features of the subsequently formed tumors. We found that tumor MVD was substantially higher in the CRC cell/MSC group. In conclusion, we demonstrated that in co-culture of MSCs and CRC cells, IL-8 secreted by MSCs is principally involved in promoting angiogenesis in CRC.

Recent cancer therapy studies have suggested that disturbing tumor-stroma interactions may help improve treatment efficacy [36]. The idea of blocking tumor angiogenesis as a cancer therapy strategy has achieved prominence in recent decades. In physiological contexts, such as development, wound healing and pregnancy, normal vascular remodeling is sustained by a balance of pro-angiogenic and anti-angiogenic signals. However, under pathological conditions, such as cancer, the tumor environment tilts toward to pro-angiogenic signals to sustain an adequate blood supply [3]. Tumor angiogenesis is influenced by numerous signaling molecules in the tumor microenvironment [37]. Several anti-angiogenic therapies directed at these molecules have been approved by the Food and Drug Administration for treating cancer, such as the humanized antibody Avastin (bevacizumab), which targets VEGF-A, and the tyrosine kinase inhibitor sorafenib, which targets Raf and VEGF and PDGF receptors [38, 39]. However, clinical trials of these drugs have not met early expectations, possibly reflecting the fact that tumor angiogenesis is controlled by multiple factors, some of which likely remain unidentified [40]. IL-8, which is also a pro-angiogenic factor, has only recently come to be considered a therapeutic target. IL-8 levels can also be a prognostic factor, reflecting adenoma-carcinoma transition and metastatic potential [41, 42]. Considerable evidence supports the hypothesis that IL-8 promotes CRC progression [43, 44], and IL-8 pathways in cancer cells have been well documented [9]. Accordingly, inhibiting IL-8 signaling in tumor cells could have therapeutic potential in modulating disease progression [45]. For example, SCH-527123, a CXCR2 antagonist, blocks tumor angiogenesis and proliferation and sensitizes tumor cells to chemotherapy [46]. These observations suggest that new therapeutic approaches directed towards IL-8 will likely have their greatest curative effect if used in conjunction with other monoclonal antibodies [45].

In summary, our results show that MSCs can stimulate CRC angiogenesis and proliferation. This pro-angiogenic effect is likely primarily increased by IL-8, and the paracrine action of MSC-derived IL-8 is dominant compared with the autocrine action of CRC cell-produced IL-8. Moreover, the rapid growth of tumors in nude mice models is likely due, at least in part, to IL-8 secreted by MSCs. Thus, by disrupting tumor-stroma interactions, suppressing the secretion of IL-8 by MSCs may provide a novel approach for CRC treatment.

## MATERIALS AND METHODS

### Reagents and antibodies

IL-8 was purchased from Prospec (USA). The DyNAmo ColorFlash SYBR Green qRT-PCR kit was purchased from Thermo Fisher Scientific (Rutherford, NJ, USA). qRT-PCR primers were designed using Oligo 7 and synthesized by Invitrogen (Carlsbad, CA, USA). CCK-8 assay kits were purchased from Dojin Laboratories (Kumamoto, Japan). Antibodies specific for CD31, Ki-67, and IL-8 were purchased from Cell Signaling Technology (USA).

### Cell culture

MSCs were isolated from bone marrow aspirates of healthy volunteers after receiving ethics approval and informed consent. To demonstrate the multipotency of shIL-8-MSCs, we cultured cells under conditions that promote differentiation into osteogenic or adipogenic lineages. Culturing in osteogenic medium for 2 weeks induced the differentiation of MSCs into osteoblasts, as confirmed by strong Alizarin Red S staining. Similarly, Oil Red O staining revealed the presence of lipid droplets in the cytoplasm of differentiated MSCs on day 21 of adipogenic differentiation. In addition, PCR results confirmed that shIL-8-MSCs had the ability to differentiate into chondrocytes and adipocytes (Supplementary Figure S1). The culture medium was 90% low-glucose Dulbecco's Modified Eagle Medium (DMEM; Hyclone, Logan, UT, USA) supplemented with 10% fetal bovine serum (FBS; Invitrogen), 100 IU/mL penicillin (Hyclone), and 100 μg/mL streptomycin (Hyclone). HUVECs were purchased from American Type Culture Collection (ATCC; Rockville, MD, USA) and cultured in Endothelial Growth Medium-2 (EGM-2; Lonza, USA). HUVECs were assayed at passages 4–6. Human CRC cell lines (SW480, LS174T and HT29) were obtained from ATCC and grown in high-glucose DMEM supplemented with 10% FBS.

### RNAi transfection

IL-8 expression was knocked down in MSCs by transduction of a lentiviral vector expressing an shRNA with the sequence 5′-CAAGAGAATATCCGAACTTTA-3′. Lentiviruses were obtained by transfection of 293T cells. MSCs at passage 2 or 3 were seeded into 6-well plates and transfected with IL-8 shRNA using X-tremeGENE HP reagent (Roche). Before experiments, GFP-positive cells were purified by flow cytometry.

### RNA sequencing

MSCs were treated with recombinant IL-1β (30 ng/mL) and TNF-α (20 ng/mL) for 12 h (*n* = 3). Then, RNA library preparation and sequencing were performed as recommended by the manufacturer (Genome Analyzer IIx; Illumina). Sequencing data were processed using Consensus Assessment of Sequence and Variation (CASAVA, version 1.8.2; Illumina) using the default settings. In brief, clusters were located using the raw images; cluster intensity and position parameters were obtained as output; and the noise for each cluster was estimated. The program determines the base sequences read from each cluster, the confidence level for each base, and whether the read passed filtering. The resulting bcl files were converted into fastq.gz files. Sequence reads were mapped to transcripts annotated in the National Center for Biotechnology Information database and used to calculate overall gene expression in terms of RPKM (reads per kilobase of exon per million mapped reads).

### qRT-PCR analyses

Total mRNA from tumor tissues or cells was extracted using an RNeasy mini kit (Qiagen) and complementary DNA (cDNA) was synthesized using a Quantitect Reverse Transcription kit (Qiagen) according to the manufacturers' protocols. Target mRNA levels were quantified by performing qRT-PCR with a DyNAmo ColorFlash SYBR Green qRT-PCR kit, as described by the manufacturer, using the following primer pairs: human IL-8, 5′-CTG GCC GTG GCT CTC TTG-3′ (forward) and 5′-CCT TGG CAA AAC TGC ACC TT-3′ (reverse); human Ki-67, 5′-CTT CCA GCA GCA AAT CTC A-3′ (forward) and 5′-ACA ATC AGA TTT GCT TCC GA-3′ (reverse); human PCNA, 5′-AGG CAC TCA AGG ACC TCA TCA-3′ (forward) and 5′-GAG TCC ATG CTC TGC AGG TTT-3′ (reverse); human bFGF, 5′- AGG GCA GAA TCA TCA CGA AGT-3′ (forward) and 5′- AGG GTC TCG ATT GGA TGG CA-3′ (reverse); human VEGF, 5′- CTC GAT CCG CTC CTT TGA TGA-3′ (forward) and 5′- CGT TGG TGC GGT CTA TGA G-3′ (reverse); and human PDGF-BB, 5′- AGA AGA GCG ACC CTC ACA TCA-3′ (forward) and 5′- CGG TTA GCA CAC ACT CCT TTG-3′ (reverse). The thermocycling protocol for all experiments was 40 cycles of denaturation at 95°C for 10 s, annealing at 60°C for 10 s, and extension at 72°C for 30 s. Target mRNA levels were normalized to those of β-actin.

### ELISA

Commercially available ELISA kits were used for measuring IL-8 secretion in the culture media. Cells were plated in 6-well plates and incubated at 37°C for 36 h. Then, equal volumes of cell culture supernatants were collected. The quantification of IL-8 protein was determined using the Quantikine IL-8 ELISA kit Neobioscience Technology Co, Ltd (Beijing, China) according to the manufacturer's protocol. The concentration of IL-8 in culture media was determined at 450 nm using a microplate reader (Tecan Trading AG, Switzerland).

### Cell counts

CRC cells or HUVECs (1 × 10^5^ cells) were treated as indicated in the text and counted every day for 5 d. After trypsinization, cells were collected by centrifugation, washed once in phosphate-buffered saline (PBS) and resuspended in 200 μL of PBS. Cells were then stained with an equal volume of 0.4% trypan blue for 5 minutes at room temperature. Viable cells (unstained) were counted using a hemocytometer.

### Cell viability assay

Cell viability was determined using a Cell Counting Kit-8 (CCK-8) assay kit. CRC cells were plated at a density of 2 × 10^5^ cells/mL on 96-well plates in culture medium (100 μL/well). After 4 h, 10 μL of CCK-8 solution was added to the cells in 96-well plates and incubated at 37°C for 0.5–4 h. The absorbance in each well was quantified at 450 nm using a microplate reader (Tecan Trading AG, Switzerland). Cell viability was calculated according to the manufacturer's instructions. At least three experiments were performed, each tested in triplicate.

### Cell migration assay

The migration ability of HUVECs was determined using 24-well transwell chambers (8-μm-pore membrane filters; Millipore) [47]. After rehydration of the extracellular matrix (ECM) layer of the chambers by incubating in serum-free media for 2 h, a 300-μL suspension of HUVECs (1 × 10^5^ cells/mL) in serum-free medium was plated into the upper chamber. Then, the lower chamber of the transwell chamber was filled with 500 μL of conditioned medium from CRC cells only, CRC cell/MSC co-cultures, or CRC cell/shIL-8-MSC co-cultures. After incubating the plates for 6 h, HUVECs that had not migrated were gently removed from the upper surface of the filters with a cotton-tipped swab, and HUVECs that had migrated through to the lower chamber were stained using the polyanionic dye Calcein AM from the LIVE/DEAD Cell Viability Assay (Invitrogen), which produces a green fluorescence in live cells. Images were acquired under a fluorescence microscope. Cell counts are expressed as the mean number of cells per field of view. The counts of migrated cells were quantified from at least five randomly selected fields from three independent experiments at 200 x magnification by three independent observers.

### *In vitro* tube-formation assay

HUVEC tube-formation assays were performed as previously described [48]. Briefly, 200 μL of growth factor-reduced Matrigel (BD Biosciences), diluted 1:1 on ice with cold DMEM, was loaded into each well of a 24-well plate and allowed to polymerize for 30 minutes at 37°C. After culturing HUVECs with conditioned media for 24 h, 5 × 10^4^ cells were added to each well, and the cells were observed under a bright-field microscope after incubation at 37°C in 5% CO_2_ for 4 h. Tube formation was evaluated by quantifying tube lengths and numbers using the Angiogenesis Analyzer module in the ImageJ toolkit.

### Xenograft studies

The effect of MSCs on CRC growth and angiogenesis *in vivo* was evaluated using a subcutaneous xenograft tumor model, established by injecting the flanks of 4-week-old male nude athymic mice with CRC cells (2 × 10^6^) mixed with or without MSCs or shIL-8-MSCs (1:1, 2 × 10^6^ each). Five mice were used in each group. Tumor volumes were calculated using the formula: tumor volume (mm^3^) = 0.5 × width^2^ × length. After 4 weeks, the mice were euthanized, and tumors were excised for weighing and subsequent experimentation. All animal studies were performed according to the guidelines of the Sun Yat-sen University Institutional Animal Care and Use Committee.

### Immunofluorescence

For cultured CRC cells, the samples were grown on 24-well plates and cultured with conditioned medium as indicated. After 48 h, the cells were fixed in 3.7% formaldehyde for 20 min, permeabilized in 0.2% Triton X-100 for 10 min, and incubated with a primary antibody against Ki-67 overnight at 4°C. The cells were then incubated with Alexa Fluor-594–conjugated secondary antibody for 1 h in the dark, after which, the nuclei were stained with DAPI (4′, 6-diamidino-2-phenylindole) for 10 min. Images were acquired using a fluorescence microscope (IX-81; Olympus Corp., Tokyo, Japan).

Tumor samples from nude athymic mice were fixed in 3.7% paraformaldehyde and then embedded in OCT compound for subsequent sectioning. Each tissue section was incubated overnight at 4°C with a primary antibody against CD31. Alexa Fluor-594–conjugated secondary antibody was added, and the sections were incubated for 1 h in the dark. The nuclei were stained by incubating with DAPI for 10 min. Images were acquired using an LSM710 confocal microscope (Zeiss). For each group, four randomly chosen high power fields per tumor (*n* = 5) were analyzed.

### Immunohistochemistry

Paraffin-embedded tumor specimens were sectioned to 4-μm thick samples. Each tissue section was deparaffinized, rehydrated, and then incubated with fresh 3% hydrogen peroxide in methanol for 25 minutes. The antigen retrieval was achieved by microwave treatment in 0.01 mol/L sodium citrate buffer (pH 6.0) at 100°C for 15 minutes. Next, nonspecific binding was blocked with normal goat serum for 10 minutes at room temperature, followed by incubation at 4°C overnight with respective primary antibodies. The slides were incubated for 10 minutes at room temperature with biotin-conjugated secondary antibodies, followed by incubation with streptavidin-conjugated peroxidase working solution for 10 minutes. Subsequently, sections were stained for 3 to 5 minutes with 3, 39-diaminobenzidine tetrahydrochloride, counterstained with Mayer's hematoxylin, dehydrated, and mounted.

### Statistical analyses

All experiments were performed at least three separate times. Outcome measures were expressed as the mean ± SEM. Comparisons among/between groups were performed using a one-way analysis of variance (ANOVA) or Student's *t*-test. CCK-8 and tumor growth curves were tested using repeated ANOVA followed by post-hoc test. *P*-values < 0.05 were considered significant.

## SUPPLEMENTARY FIGURES


