# Supplementary material for: Bone marrow-derived mesenchymal stem cell-secreted IL-8 promotes the angiogenesis and growth of colorectal cancer
Source: Oncotarget. 2015 Oct 23;6(40):42825–37. doi: 10.18632/oncotarget.5739 (PMC4767474; doi:10.18632/oncotarget.5739)
Supplement: Supplementary file 1 [file oncotarget-06-42825-s001.pdf]

SUPPLEMENTARY FIGURES

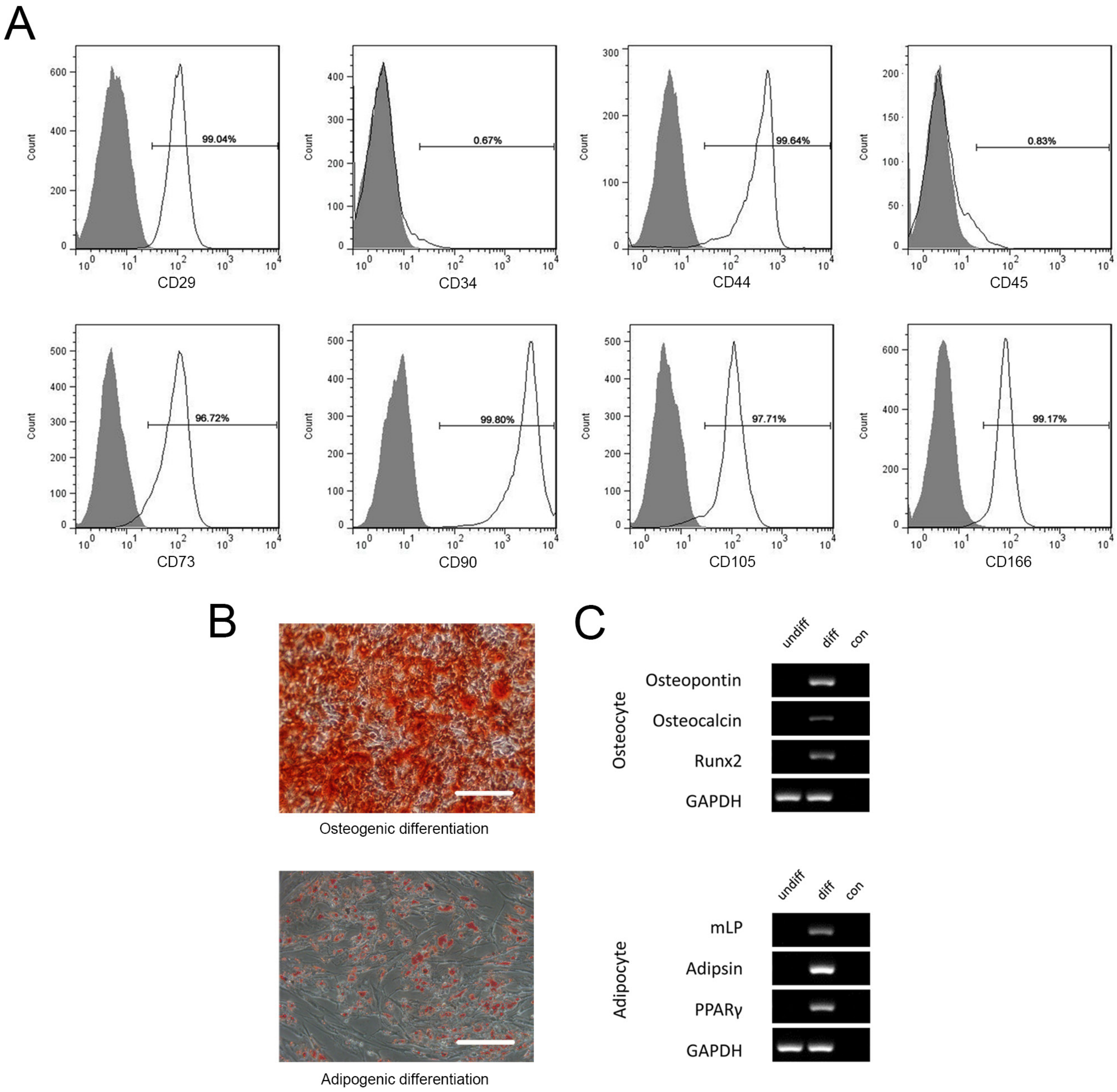

Supplementary Figure S1: Characterization of shIL-8-MSCs. A. CD29, CD34, CD44, CD45, CD73, CD105, and CD166 expression in shIL-8-MSCs were analyzed by flow cytometry, which showed that transduced cells maintained the phenotype of MSCs. **B.** MSCs were cultured in osteogenic medium for 2 weeks. Differentiation of MSCs into osteoblasts was confirmed by Alizarin Red S staining. Similarly, differentiation of MSCs into adipocytes was confirmed by Oil Red O staining, which showed lipid droplets in the cytoplasm of differentiated MSCs. Scale bar, 100 μm. **C.** MSCs were cultured as shown in (B). Osteogenic and adipogenic markers were detected by PCR.

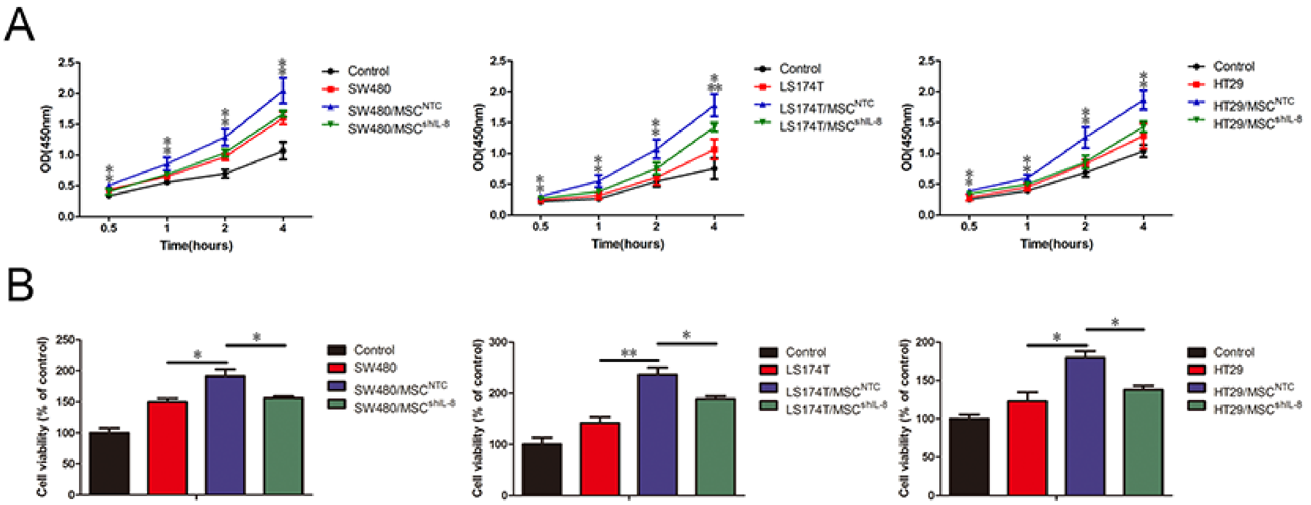

Supplementary Figure S2: MSCs promote endothelial cell proliferation and migration through IL-8 secretion. A. & B. HUVECs were cultured with conditional medium from CRC cells, CRC cell/MSC co-cultures, or CRC cell/shIL-8-MSC co-cultures. Cell viability was measured using CCK-8 assays, performed as described in Materials and Methods. The results are presented as the mean values from three independent experiments (*, $p < 0.05$, **, $p < 0.01$).

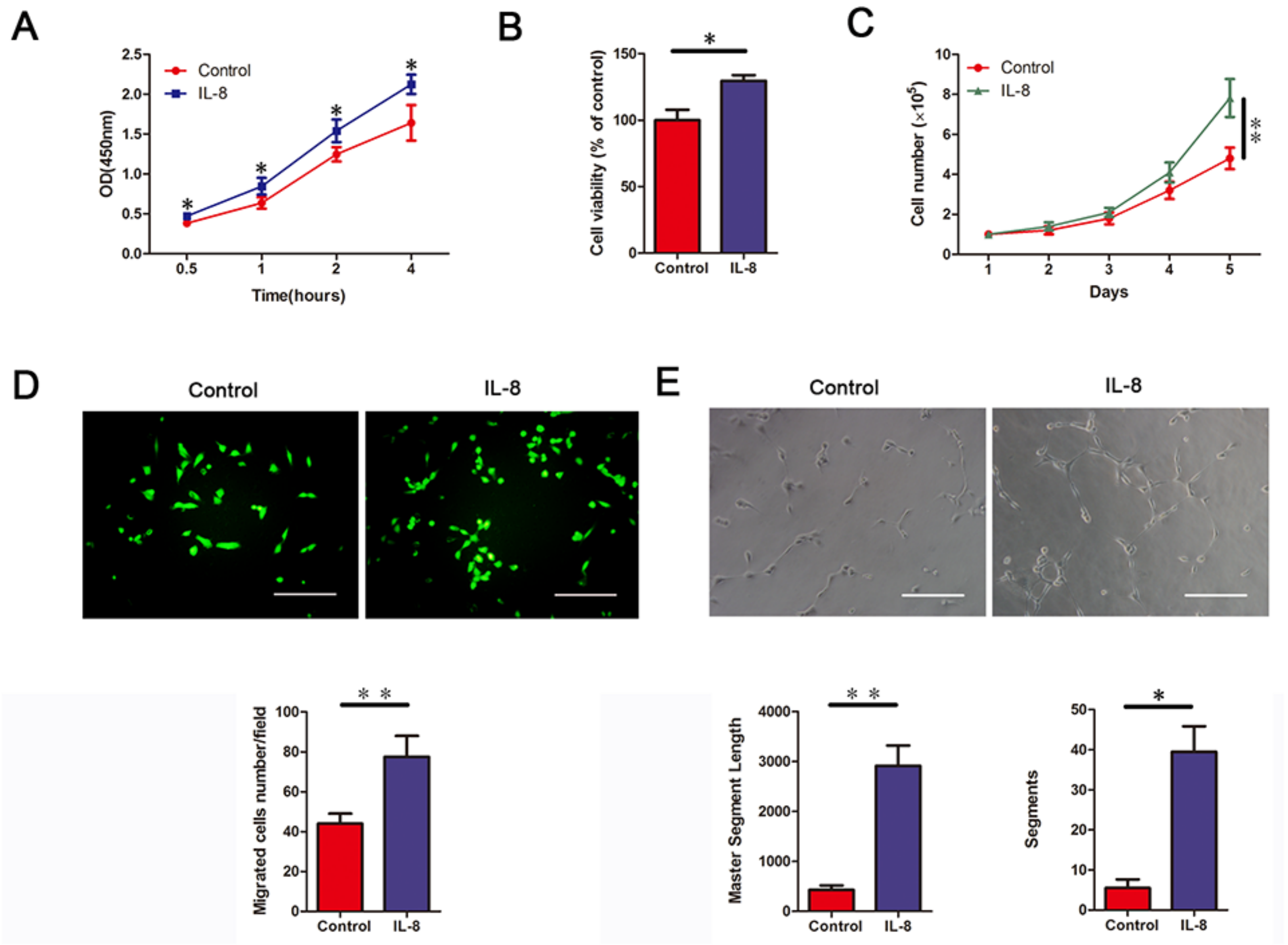

Supplementary Figure S3: IL-8 promotes endothelial cell proliferation, migration and tube formation. A. & **B.** IL-8 (100 ng/mL) was added to the culture medium of HUVECs. HUVEC viability was determined by CCK-8 assay. The results are presented as the mean values from three independent experiments (*, $p < 0.05$). **C.** The numbers of HUVECs following culture with medium alone or medium containing IL-8 (100 ng/mL) were determined. The counts of the cells are presented as the mean values from three independent experiments using a hemocytometer (**, $p < 0.01$). **D.** Assay of HUVEC migration. HUVECs that migrated through the transwell membrane were stained with Calcein AM. Scale bar, 100 μm. Migrated HUVECs were counted. The counts of the cells are presented as the mean values per field from at least five randomly selected fields from three independent experiments (**, $p < 0.01$). **E.** HUVECs were stimulated by IL-8 (100 ng/mL) for 4 h. Images showed tube-formation ability of HUVECs before and after IL-8 stimulation. Scale bar, 100 μm. Capillary-like structures were analyzed by the Angiogenesis Analyzer module in the Image J toolkit. The results are presented as the mean values per field from at least five randomly selected fields from three independent experiments (*, $p < 0.05$, **, $p < 0.01$).

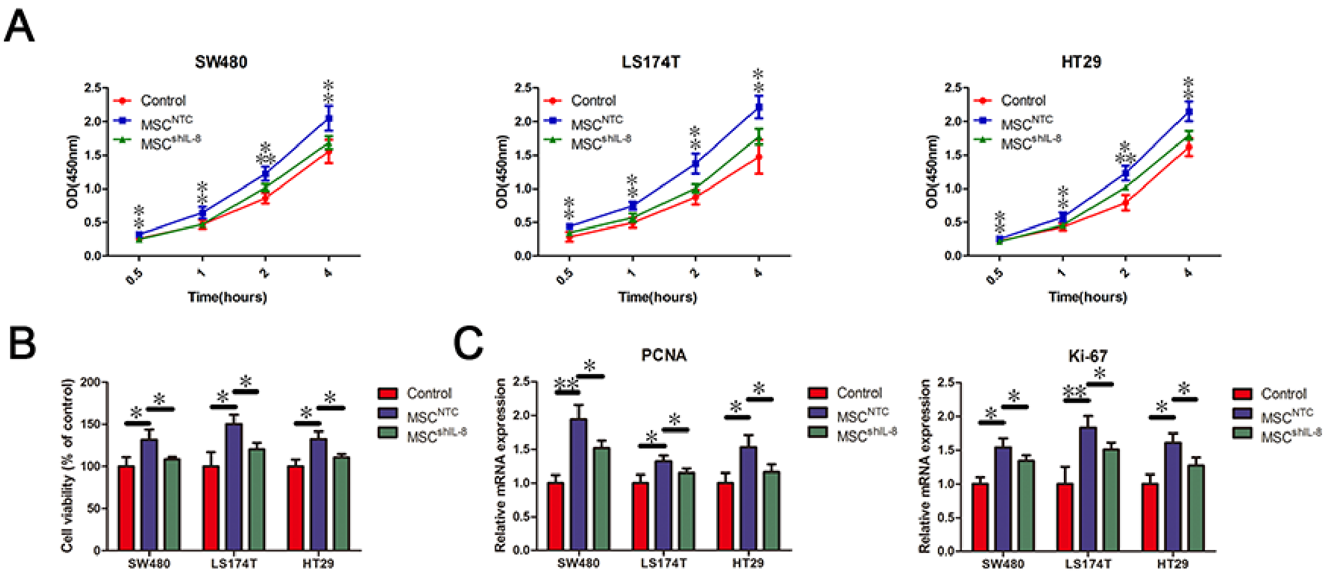

Supplementary Figure S4: MSC-secreted IL-8 stimulates CRC cell proliferation. A. & **B.** The viability of CRC cells (SW480, LS174T and HT29) cultured with fresh medium or conditioned medium from CRC cell/MSC co-cultures or CRC cell/shIL-8-MSC co-cultures was measured by CCK-8 assay. The results are presented as the mean values from three independent experiments (*, $p < 0.05$, **, $p < 0.01$). **C.** CRC cells were cultured with fresh medium or conditioned medium from CRC cell/MSC co-cultures or CRC cell/shIL-8-MSC co-cultures. PCNA and Ki-67 mRNA levels was measured by qRT-PCR (*, $p < 0.05$, **, $p < 0.01$).

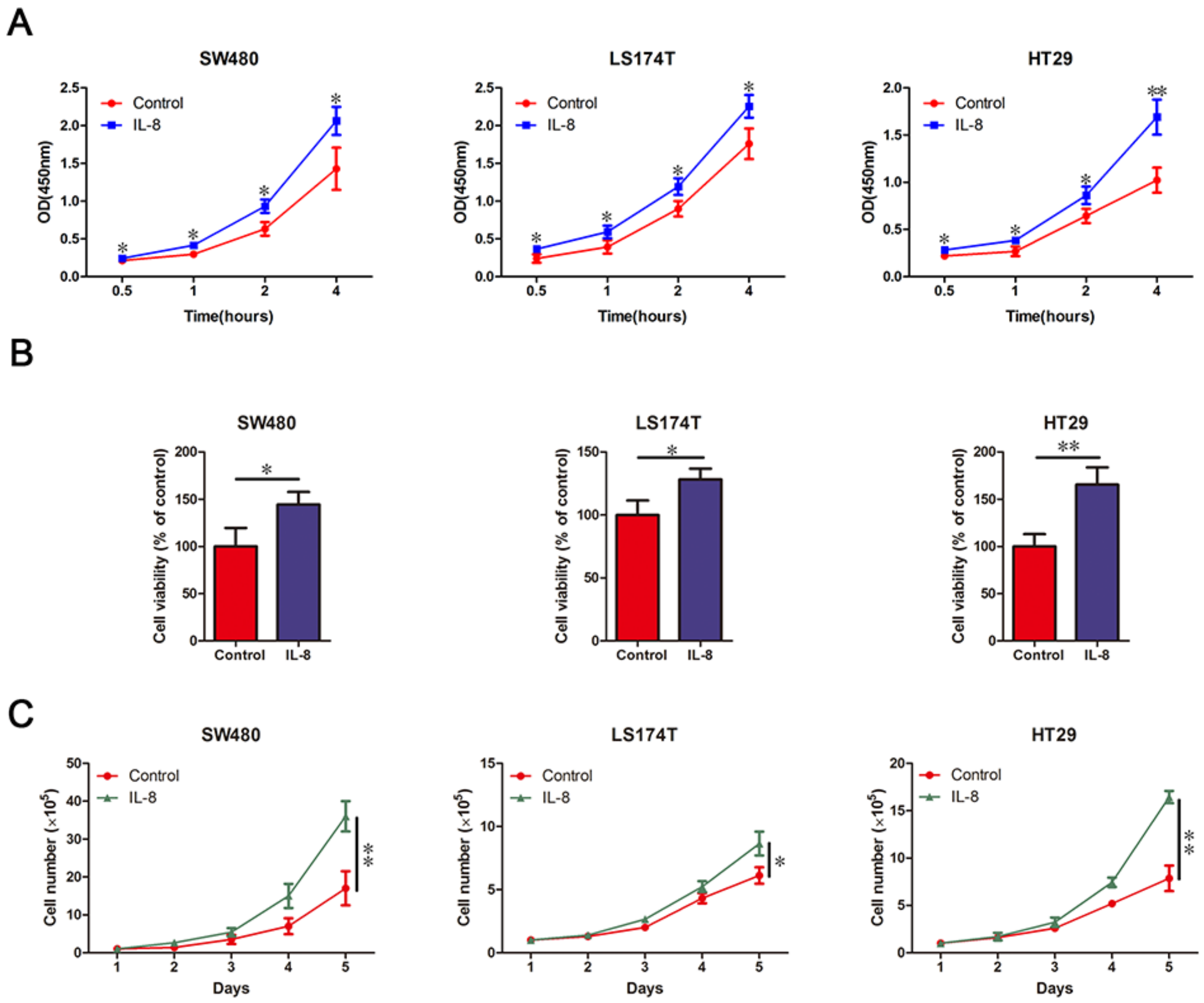

Supplementary Figure S5: IL-8 induces CRC cell proliferation. A. & B. The viability of CRC cells following stimulation with IL-8 (100 ng/mL) was determined by CCK-8 assay. The results are presented as the mean values from three independent experiments (*, $p < 0.05$, **, $p < 0.01$). **C.** The numbers of CRC cells (SW480, LS174T and HT29) following stimulation with IL-8 (100 ng/mL) were counted. The counts of the cells are presented as the mean values from three independent experiments using a hemocytometer (*, $p < 0.05$, **, $p < 0.01$).
